# *Ipomoea pes-caprae IpASR* Improves Salinity and Drought Tolerance in Transgenic *Escherichia coli* and *Arabidopsis*

**DOI:** 10.3390/ijms19082252

**Published:** 2018-08-01

**Authors:** Jie-Xuan Zheng, Hui Zhang, Hua-Xiang Su, Kuai-Fei Xia, Shu-Guang Jian, Mei Zhang

**Affiliations:** 1 Key Laboratory of South China Agricultural Plant Molecular Analysis and Genetic Improvement, South China Botanical Garden, Chinese Academy of Sciences, Guangzhou 510650, China; zhengjiexuan16@mails.ucas.ac.cn (J.-X.Z.); zhanghuir@mails.ucas.ac.cn (H.Z.); suhuaxiang17@mails.ucas.ac.cn (H.-X.S.); xiakuaifei@scbg.ac.cn (K.-F.X.); 2 University of the Chinese Academy of Sciences, Beijing 100039, China; 3 Key Laboratory of Applied Botany, South China Botanical Garden, Chinese Academy of Sciences, Guangzhou 510650, China; jiansg@scbg.ac.cn

**Keywords:** *Ipomoea pes-caprae* L., *IpASR*, salt, drought

## Abstract

*Ipomoea pes-caprae* L. is an extremophile halophyte with strong adaptability to seawater and drought. It is widely used in the ecological restoration of coastal areas or degraded islands in tropical and subtropical regions. In this study, a new *abscisic acid*, *stress* *and* *ripening* (*ASR*) gene, *IpASR*, was reported, and is mainly associated with biological functions involved in salt and drought tolerance. Sequence analysis of IpASR showed that this protein contains an ABA/WDS (abscisic acid/water deficit stress) domain, which is a common feature of all plant ASR members. Overexpression of *IpASR* improved *Escherichia coli* growth performance compared with the control under abiotic stress treatment. The transgenic overexpressing *IpASR Arabidopsis* showed higher tolerance to salt and drought stress than the wild type and lower accumulation of hydrogen peroxide (H_2_O_2_) and superoxide (O_2_^−^) accompanied by increased antioxidant enzyme activity in vivo. IpASR exhibits transcription factor’s activity. Therefore, the overexpression of *IpASR* in *Arabidopsis* is supposed to influence the expression of some genes involved in anti-oxidative and abiotic stresses. The results indicate that *IpASR* is involved in the plant response to salt and drought and probably acts as a reactive oxygen species scavenger or transcription factor, and therefore influences physiological processes associated with various abiotic stresses in plants.

## 1. Introduction

Due to the immobility of plants, their growth and development is frequently influenced by external environmental signals, particularly by certain biotic or abiotic stresses. The response of plants to stress mainly involves the following steps: The perception and transduction of stress signals and the induction of the expression or synthesis of the response gene or protein, resulting in cellular changes in physiological and biological characteristics, eventually decreasing the damage caused by stress [1]. Among these responses, the regulation of the expression of stress-related genes is a vital step for the survival or tolerance of plants in adverse conditions. The isolation and characterization of stress-tolerance genes provides a foundation for the genetic improvement of plants and crops [2]. *ASR* (*abscisic acid*, *stress*, *ripening-induced*) genes are plant-specific small gene families that are mostly involved in the response to abiotic (mainly drought and salinity) stresses and fruit ripening [3]. The adaptation of many plants and crops to stressful environments in the context of environmental degradation and climate change is being increasingly studied in an attempt to improve stress tolerance and isolate candidate stress-related genes for plant genetic engineering.

ASR proteins occur in many plant species and belong to a family of hydrophilic proteins that have important roles in the response to abiotic stresses. All ASRs harbor the ABA/WDS (abscisic acid/water deficit stress) domain (Pfam entry PF02496) as a common denominator, indicating that they might have similar biological functions in the response of plants to dehydration or the abscisic acid (ABA) signaling pathway [4]. Since ASRs may not only function as transcription factors, but also as intrinsically unstructured chaperones possessing similar plant LEA (late embryogenesis abundant) protein characteristics, some reporters classified as ASR proteins have been placed into the LEA protein group because of their small size, physicochemical properties, and participation in stress pathways [5]. While with a distinguished feature from LEA family, a large multigene family, the ASR family is obviously absent from Brassicaceae plants (including *Arabidopsis*), and usually comprise few members in other plants. The first member of *ASR*, tomato *Asr1*, was identified by screening a tomato fruit cDNA library using a cDNA differential hybridization approach, with accumulated transcripts observed in the ripening fruit, water-stressed leaves, or under ABA treatment, hence its name [6]. Following this, a fairly large number of *ASR* orthologs were characterized from many plant species along with the advance of the Plant Genome Project [7,8,9,10].

Plant-specific *ASR* genes are typically upregulated by a wide range of factors, including drought, cold, salt, ABA, and injury, in addition to the plant response to developmental and environmental signals. Drought and salinity are two common abiotic stressors that often challenge plant growth and development, thereafter triggering complex multicomponent signaling pathways to restore cellular homeostasis and promote plant survival [11]. Recent studies in many plants suggest that different *ASRs* could elevate the tolerance of transgenic plants to high salinity and dehydration. For instance, the *MpAsr* from *Musa paradisiaca* (banana) conferred improved osmotic tolerance to transgenic *Arabidopsis* [12]. *Salicornia brachiata* is an extreme halophyte that can survive in salty marshes in coastal areas. *SbASR-1* from *S. brachiata* conferred enhanced salinity and drought endurance in transgenic tobacco and groundnut [13,14]. *Suaeda liaotungensis* is also an extreme halophyte that is mainly distributed on saline and alkaline lands in Heilongjiang province and the Inner Mongolia province in China. The *SlASR* from *S. liaotungensis* also elevates salinity and drought tolerance in transgenic plants when overexpressed in *Arabidopsis* [15]. The gramineous plants *Setaria italica* and *Brachypodium distachyon* are both drought resistant plants. *SiASR4* [16] from *S. italica* and *BdASR1* [9] from *B. distachyon* both enhanced salt and drought tolerance when they were separately overexpressed in *Arabidopsis* or tobacco.

*Ipomoea pes-caprae* L. (Convolvulaceae) is a wild perennial lianaceous herb that naturally occurs in seashore areas of tropical or subtropical regions worldwide. It exhibits high nutrition uptake and utilization efficiency and strong resistance to salt and drought, and hence has been used as the first “green barrier” for sand fixation, island-greening, and the ecological restoration of coastal regions and islands and reef construction [17]. As evidenced by its habitat, *I. pes-caprae* is also an extreme halophyte, which is considered to be the best resource for characterizing stress-tolerance genes and promoters [18]. These are then ultimately introduced into non-halophytic crop species to improve their performance under saline conditions [19]. In this study, we mainly focused on the characterization of *IpASR* from *I. pes-capraei*, and performed transgenic assays in *Escherichia coli* and *Arabidopsis thaliana* to functionally validate this gene under salt/drought and other abiotic stress conditions.

## 2. Results

### 2.1. IpASR Encodes a Protein with an ABA/WDS Domain

*IpASR* was obtained by screening the *I. pes-caprae* full-length cDNA library (data not published). The full-length cDNA of *IpASR* is 962 bp with a 648 bp open reading frame (ORF) encoding a protein of 215 amino acids. The sequences of *IpASR* cDNA and protein are listed in Supplementary Materials Table S1. PCR amplification of genomic sequence of *IpASR* indicated that this gene contains a 192 bp intron (Supplementary Materials Table S1). The ProtParam-calculated molecular weight of IpASR was 24.57 kDa with a theoretical isoelectric point (pI) of 5.42. Glutamic acid was the most abundant amino acid in the IpASR protein sequence (encompassing 17.67% of the total number of amino acids), followed by lysine (11.63%), and glycine (11.16%). Furthermore, the IpASR sequence carried 49 negatively charged (aspartic acid and glutamic acid) and 31 positively charged (arginine and lysine) residues. The instability index (II) of IpASR was also assessed by ProtParam, which classified this protein as stable with an instability index (II) score of 36.82 (<40). The negative value (−1.637) of the grand average of hydropathicity (GRAVY) indicated that the IpASR protein was hydrophilic. The PHYRE^2^ program suggested that 77% of the amino acid residues of IpASR were disordered, indicating that IpASR is an intrinsically disordered protein (IDP). The properties of the deduced IpASR are listed in Table 1.

**Figure 1:**
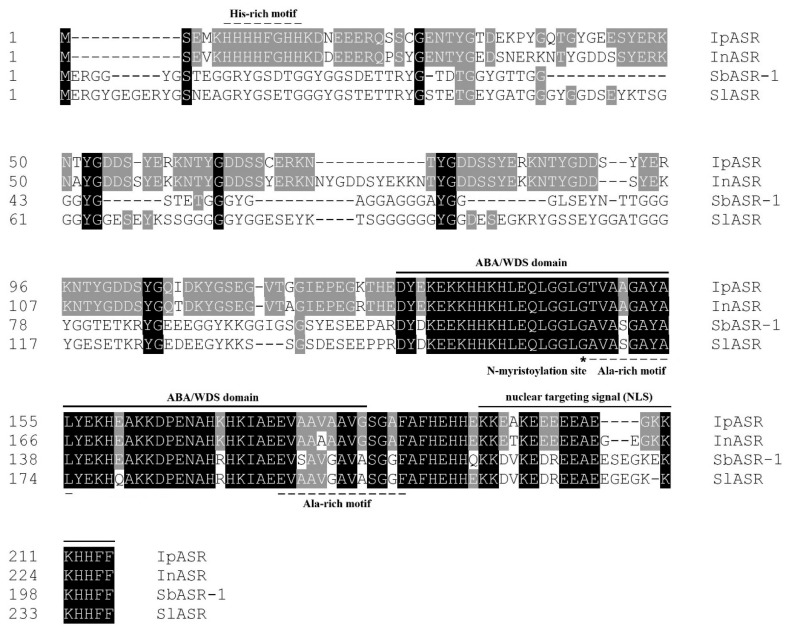
Comparison of IpASR from *I. pes-caprae* with abscisic acid, stress and ripening (ASR) proteins from other plant species; *I. nil* InASR (BAF46301.1), *S. brachiata* SbASR-1 (ACI15208.1), and *S. liaotungensis* SlASR (AGZ20206.1). The abscisic acid/water deficit stress (ABA/WDS) domain, one His-rich motif, two Ala-rich motifs, and one cryptic *N*-myristoylation site were marked.

SmartBLAST analysis with the amino acid sequence of IpASR showed that it is highly homologous to InASR from *Ipomoea nil*, SdASR-1 from *S. brachiata*, and SlASR from *S. liaotungensis* (Figure 1). IpASR contains a highly conserved ABA/WDS functional domain (amino acids 128–184). At the N-terminal, a consensus sequence containing six His residues in IpASR was identified, which is speculated as a metal binding His-rich motif. There are also two Ala-rich motifs at the N-terminal region, one site for a cryptic *N*-myristoylation, and a putative nuclear localization signal (NLS), which implied that IpASR belongs to nucleoproteins (Figure 1).

### 2.2. Phylogenetic Analysis

**Figure 2:**
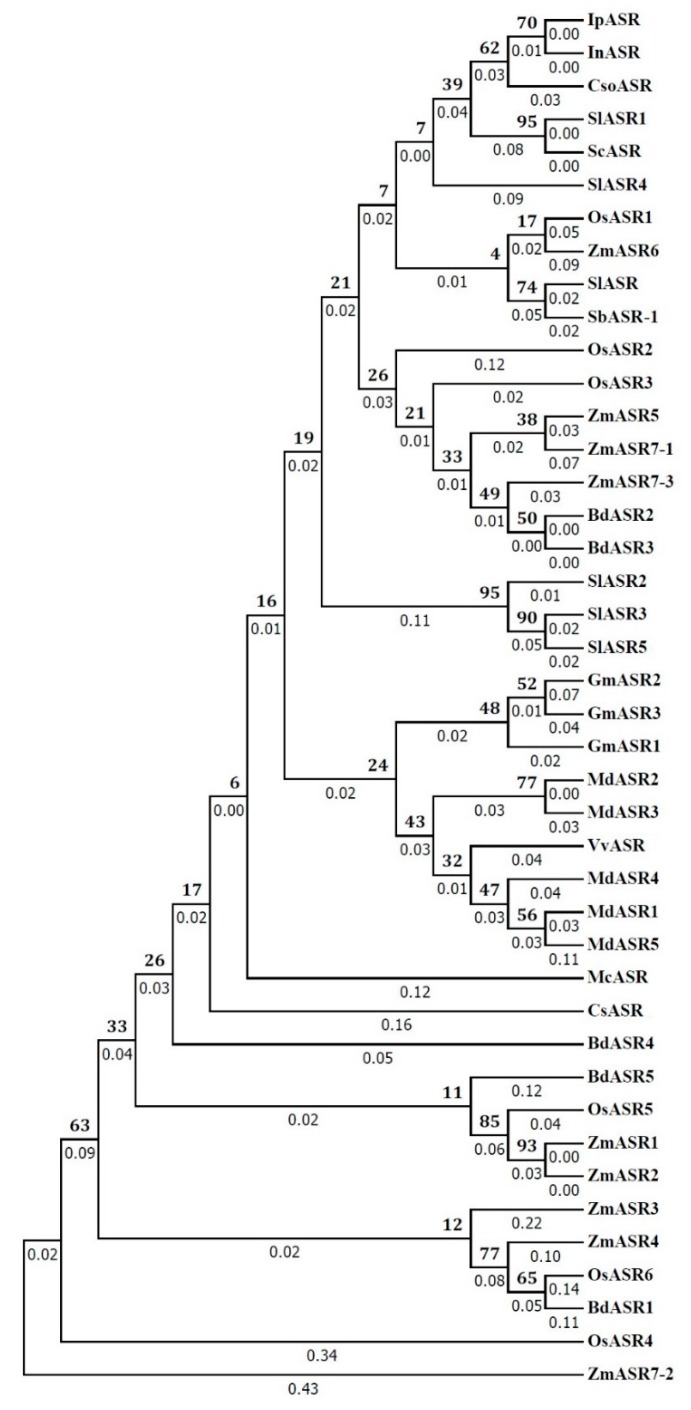
Phylogenetic analysis of IpASR with other ASR proteins. GenBank accession numbers are listed in the “Materials and Methods” (Section 4).

The deduced amino acid sequences of IpASR1 and the other 41 ASR members from other plant species were studied using phylogenetic analysis (Figure 2). Based on the amino acid sequences, IpASR shared maximum identity with the ASR proteins of morning glory InASR (Accession no. BAF46301.1) and *Calystegia soldanella* CsoASR (Accession no. BAB19963.1), which both belong to Convolvulaceae. However, the phylogenetic tree did not reflect the evolutionary relationships between glycophyte and halophyte or monocot and dicot.

### 2.3. Expression Profiles of IpASR

**Figure 3:**
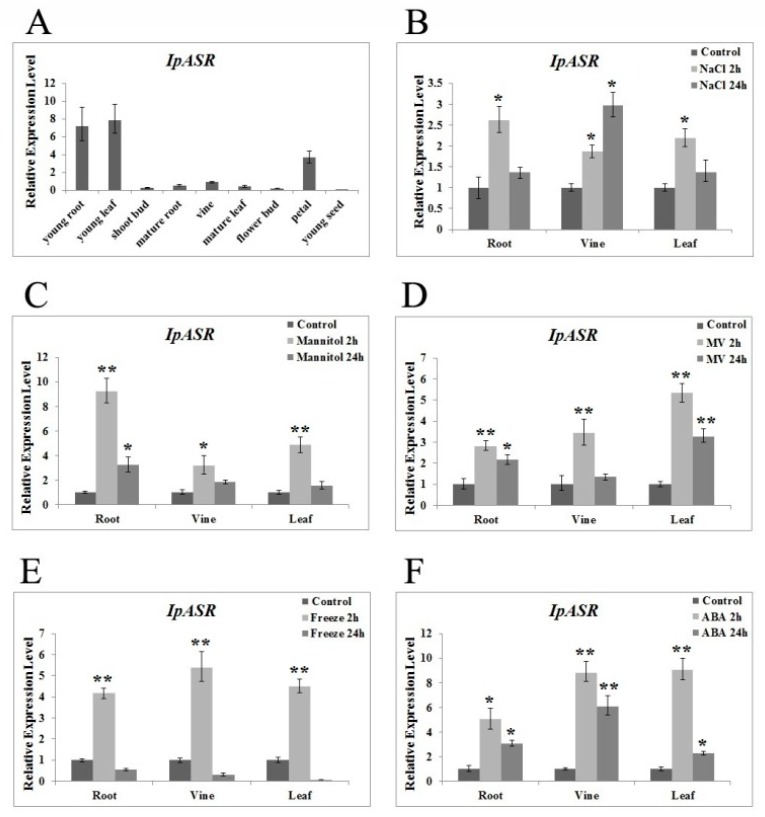
Expression profiles of the *IpASR* gene among the *I. pes-caprae* tissues. (**A**) Differential expression of *IpASR* in various tissues (young root, young leaf, shoot bud, mature root, vine, mature leaf, flower bud, petal, and young seed). Time-course expression patterns of *IpASR* in response to different abiotic stresses: NaCl (**B**) mannitol simulating dehydration (**C**) MV (methyl viologen), (**D**) freezing, (**E**) and abscisic acid (ABA) treatment. (**F**) Values are the means ± standard deviation (SD) (*n* = 3). Asterisks indicate significant differences from the control (Student’s *t*-test *p* values, * *p* < 0.05 and ** *p* < 0.01).

To investigate the expression pattern of *IpASR* in *I. pes-caprae*, qRT-PCR was performed with total RNA extracted from the various tissues. Our results demonstrated that *IpASR* was expressed constitutively in most of the *I. pes-caprae* tissues (Figure 3A). The highest level of *IpASR* transcription was detected in the young roots and young leaves, and the flower petals also showed a high expression of *IpASR*, while *IpASR* was weakly expressed in the adult plants tissues/cells.

To determine the expression of *IpASR* under various stresses, the mRNA of *IpASR* in the root, vine, and leaf under various stress treatments was also investigated (Figure 3B–F). After challenging with 300 mM NaCl and 300 mM mannitol, the transcription level of *IpASR* exhibited a slight increase in the root, vine, and leaf tissues at 2 h, and then decreased at 24 h. Low temperature (0 °C) stress also rapidly and slightly induced the expression of *IpASR*, peaking at 2 h. Additionally, we also evaluated the expression change of *IpASR* under MV (methyl viologen) and ABA treatment. Our results showed that both MV and ABA could greatly and rapidly increase the transcription level of *IpASR* (Figure 3). The expression pattern of *IpASR* indicated that the biological role of *IpASR* was involved in plant cellular growth or development, as well as the abiotic stress response.

### 2.4. IpASR Improves the Stress Tolerance of E. coli

**Figure 4:**
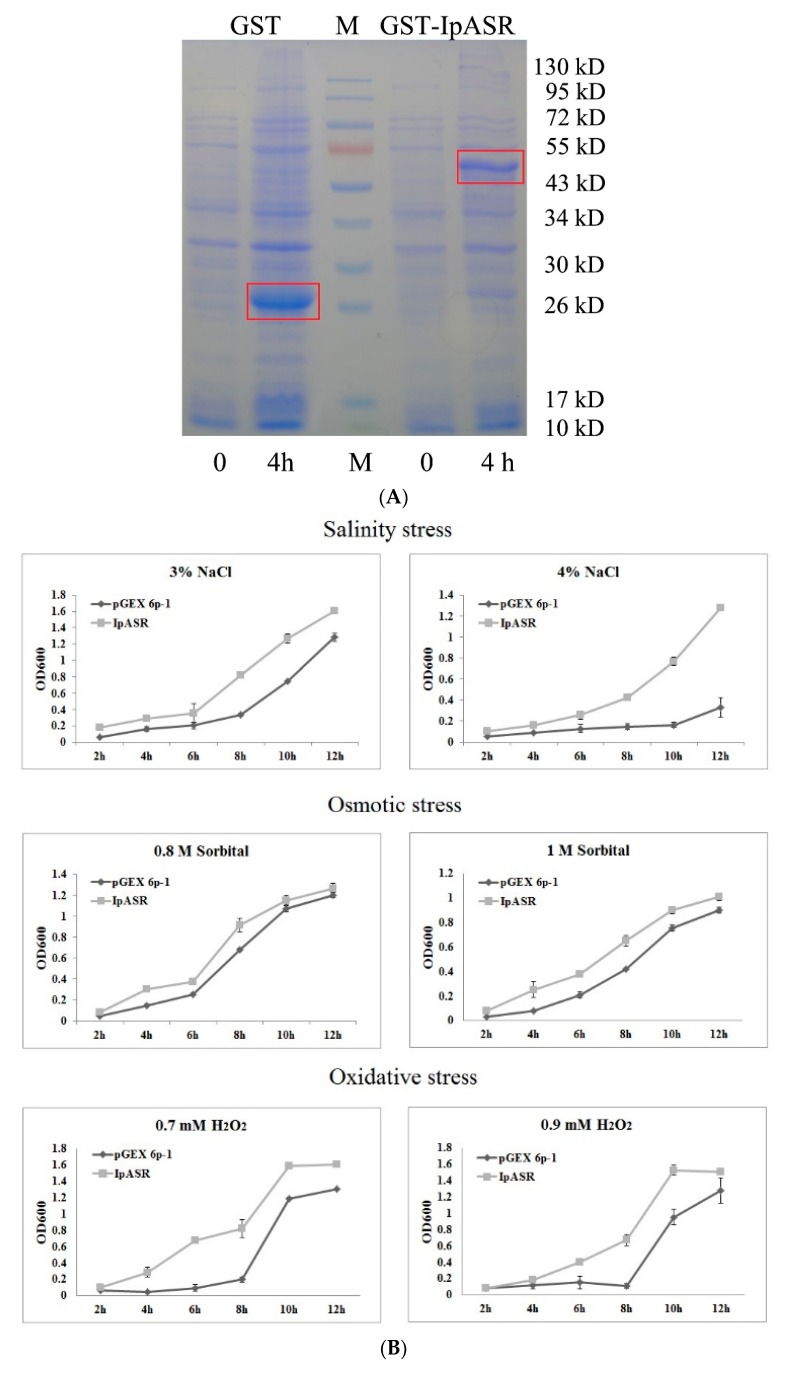

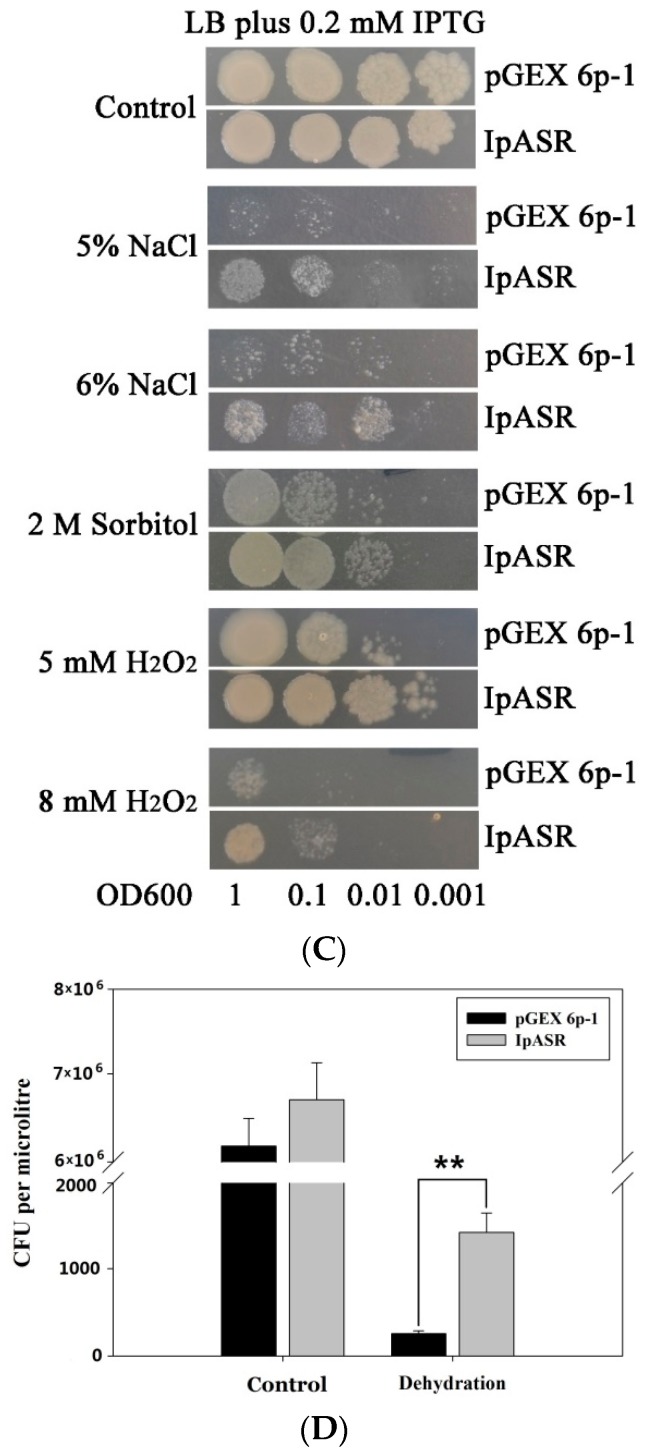
Functional analysis of IpASR induced-expression for salt, drought, and H_2_O_2_ tolerance in *E. coli*. (**A**) Induced expression of the GST-IpASR (IpASR-pGEX 6p-1) fusion protein and single GST (pGEX 6p-1 empty vector) protein in *E. coli*. 0 and 4 h: The respective IPTG (isopropyl β-d-thiogalactopyranoside) induction times. (**B**) The growth performance of *E. coli* BL21 (pGEX 6p-1, upper)/(IpASR-pGEX 6p-1) on Luria-Bertani (LB) plates containing stress factors. Control (top): LB medium; 5% NaCl: LB medium containing 5% NaCl; 6% NaCl: LB medium containing 6% NaCl; 2 M Sorbitol: LB medium containing 2 M sorbitol; 5 mM H_2_O_2_: LB medium containing 5 mM H_2_O_2_; 8 mM H_2_O_2_: LB medium containing 8 mM H_2_O_2_. The cell cultures were adjusted to OD_600_ until 1.0 and then diluted serially (1:10, 1:100, and 1:1000, respectively). Two microliters of each sample was spotted onto the LB plates containing 0.2 mM IPTG. (**C**) Growth kinetics of *E. coli* transformed with pGEX 6p-1 (control) and IpASR-pGEX 6p-1. Cells were grown until the OD at 600 nm reached 0.5, following which 0.2 mM IPTG was added, and the cells were incubated for 4 h at 30 °C. The cells were then transferred into fresh LB medium (1:100, plus 0.2 mM IPTG) supplied with different concentrations of NaCl (3% or 4%), sorbitol (0.8 or 1 M), or H_2_O_2_ (0.7 or 0.9 mM). The bacteria were cultured at 37 °C and 200 rpm. The OD_600_ values were measured every 2 h to evaluate the growth conditions. (**D**) Cell viability relates to CFU (colony former unit) before (control) and after desiccation (40 °C for 4 h). Error bars indicate the SD based on three replicates. Asterisks indicate significant differences from the control (Student’s *t*-test *p* values, ** *p* < 0.01).

The ORF sequence of the *IpASR* cDNA was subcloned into the pGEX 6p-1 vector and GST-IpASR was expressed in *E. coli* BL21 (DE3) cells. High accumulation of GST-IpASR and GST (control) proteins were observed at 4 h after inducing with isopropyl β-d-thiogalactopyranoside (IPTG). The differences in molecular weights between the fusion GST-IpASR and GST proteins confirmed the predicted 24-kDa molecular weight of the IpASR protein (Figure 4A).

The effect of IpASR accumulation on *E. coli* stress tolerance was assessed by spot and liquid culture assays. When assayed with the liquid culture method under stress conditions, the growth of *E. coli* cells expressing the GST-IpASR fusion protein increased significantly with time compared to the BL21-DE cells expressing GST only (Figure 4A). Cells harboring GST-IpASR demonstrated better performance and a quicker growth rate against NaCl (3% and 4%), sorbitol (0.8 and 1 M), and H_2_O_2_ (0.7 and 0.9 mM) stresses compared to the control *E. coli* BL21 (DE3) cells expressing GST only (Figure 4B). Comparable *E. coli* stress tolerances were observed for the spot assay, where the number of colonies was higher in the stress treatments for recombinant cells compared to *E. coli* and the vector (GST) control (Figure 4C).

The tolerance of *E. coli* with the empty vector (GST) control and GST-IpASR subjected to drought stress was also determined by spreading the culture dilutions on Luria-Bertani (LB) plates containing 0.2 mM IPTG after drying the bacterial precipitate at 40 °C for 4 h, and recovering it in 100 μL liquid LB medium for 1 h. Figure 4D shows a clear difference in the number of viable *E. coli* cells before and after desiccation stress. Before the drying process, very similar CFUs (colony former unit, expressed in ×10^6^ units) were obtained for the control (GST) and GST-IpASR-expressing cells, whereas after desiccation, although only a very small fraction of the cells survived, the number of CFUs expressing GST-IpASR was five times higher than in the control cells. This result suggests that IpASR expression in *E. coli* improved its survival capacity after desiccation.

### 2.5. IpASR Localizes to the Cell Nucleus

**Figure 5:**
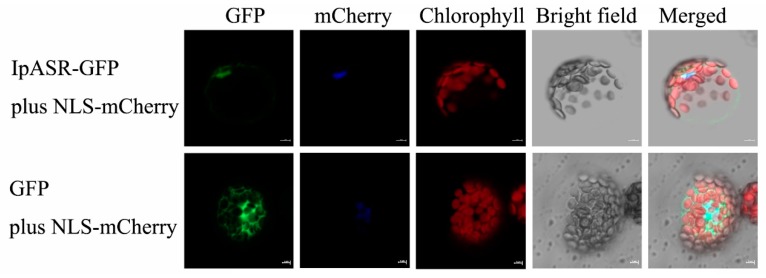
Subcellular localization of IpASR. *Arabidopsis* protoplasts expressing the IpASR-GFP fusion protein (upper) and GFP (lower) observed under a laser scanning confocal microscope. The blue color indicates the nucleus using mCherry as the nuclear marker. The red color indicates the autofluorescence emitted by chloroplasts. The bar represents 5 μm.

To investigate the subcellular localization of IpASR, a transient expression assay was performed with the IpASR-GFP fusion protein and GFP (green fluorescent protein) alone (as a control) in *Arabidopsis* mesophyll protoplasts. The fluorescence of IpASR-GFP was mainly distributed in the nucleus, with similar distribution patterns as NLS-mCherry, while the GFP was distributed throughout the protoplast cells (Figure 5). The results obtained in the protoplasts, in combination with the protein sequence analyses of IpASR (Figure 1), indicated that IpASR localized to the cell nucleus as a nuclear protein.

### 2.6. IpASR Exhibits Transcriptional Activity

**Figure 6:**
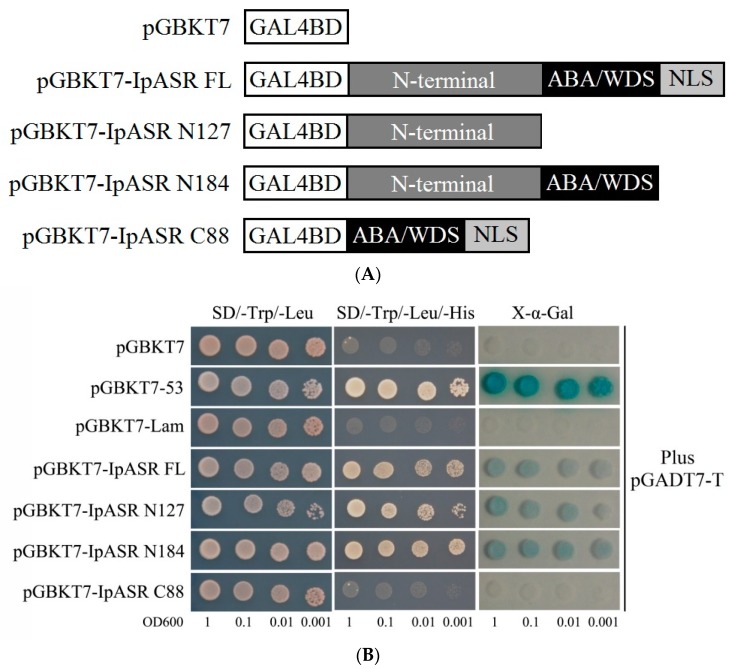
GAL4 DNA binding domain-IpASR fusion analyses for transactivation activity and α-galactosidase assay in yeast. (**A**) The GAL4 DNA binding domain was fused with different parts of IpASR and transformed into the yeast strain AH109 containing the *His*3 and *MEL*1 reporter genes. (**B**) Analysis of α-galactosidase activity of the relative yeast strains on plates. The yeast culture (OD_600_ to 1) was serially diluted to OD_600_ values of 0.1, 0.01 and 0.001, and then the 2-μL yeast liquid was spotted onto SD plates and cultured for 2 days at 30 °C.

ASRs from other plants species have been reported to possess transactivation activity [4,9]. To assess the transactivation activity of IpASR, its complete coding region was fused in-frame with the GAL4 DNA binding domain of the pGBKT7 vector in this study. Furthermore, three truncated IpASR fragments were also generated into the pGBKT7 vector with the purpose of categorizing the crucial region of IpASR for driving transcription (Figure 6A). The yeast growth on the SD medium lacking tryptophan, leucine, and histidine (SD/-Trp/-Leu/-His), and the staining assay of α-galactosidase activity in Figure 6B, indicate that the transactivation sites of IpASR are located in the N-terminal of IpASR.

### 2.7. IpASR Improves Abiotic Stress Tolerance in Transgenic Arabidopsis

**Figure 7:**
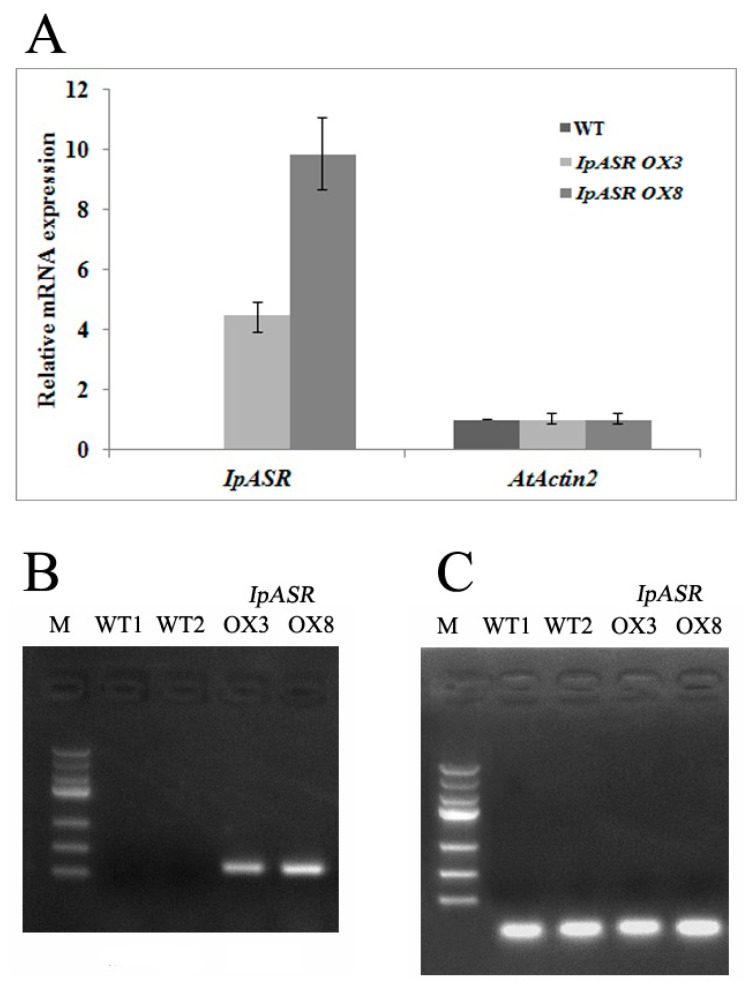
Overexpression analyses of *IpASR* in transgenic *Arabidopsis* lines (*IpASR OX3* and *IpASR OX8*). (**A**) Quantitative RT-PCR analysis of *IpASR* in transgenic *Arabidopsis* lines and wild type (WT) *Arabidopsis*. *Actin2* was used as an internal control. Error bars indicate the SD based on three replicates. (**B**) RT-PCR analysis of *IpASR* in transgenic *Arabidopsis* lines and two WT lines. (**C**) RT-PCR analysis of *AtACT2* in transgenic *Arabidopsis* lines and two WT lines as a control.

To assess the function of *IpASR* in stress tolerance, transgenic *Arabidopsis* plants overexpressing *IpASR* under the control of the CaMV35S promoter were generated. Finally, two independent homozygous *IpASR* T3 lines (*IpASR OX3* and *IpASR OX8*) with high expression levels were selected for further functional analysis (Figure 7).

**Figure 8:**
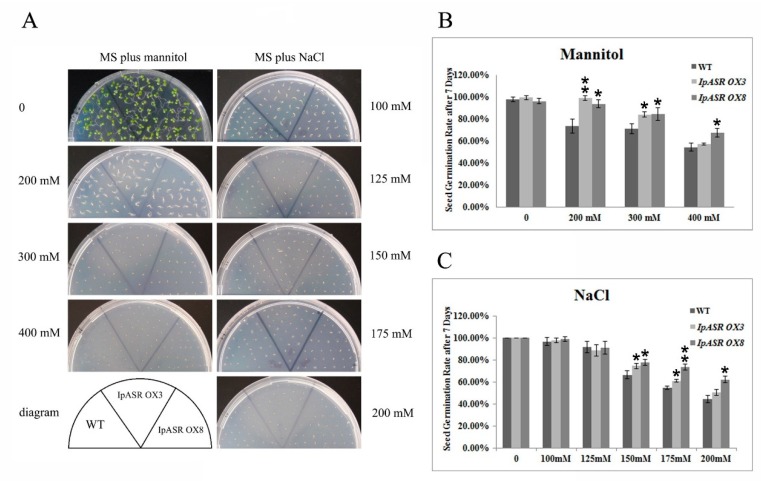
Osmotic and salt stress analyses of transgenic plants with *IpASR* with regards to seed germination rate. (**A**) Photographs of transgenic lines (*IpASR OX3* and *IpASR OX8*) and WT seeds germinated on MS (Murashige and Skoog) medium or MS medium with mannitol (left, 200, 300 and 400 mM) or NaCl (right, 100, 125, 150, 175 and 200 mM) for 7 days. (**B**,**C**) Seed germination rates in WT and transgenic lines under mannitol (**B**) and NaCl (**C**) stress after 7 days. Error bars indicate the SD based on over three replicates (*n* ≥ 3). Asterisks indicate significant differences from the control (Student’s *t*-test *p* values, * *p* < 0.05 and ** *p* < 0.01).

**Figure 9:**
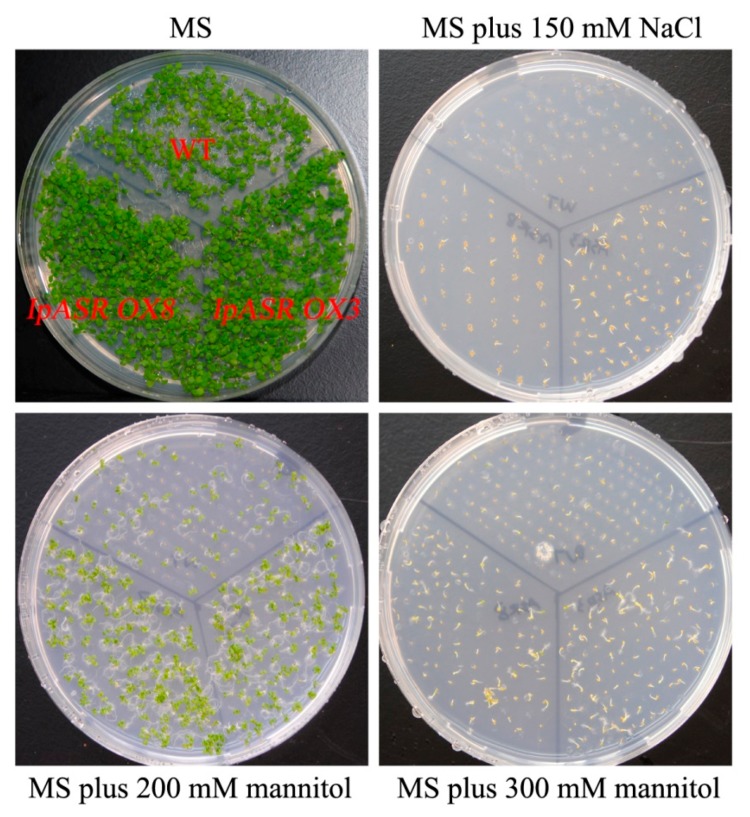
Photographs of transgenic lines (*IpASR OX3* and *IpASR OX8*) and WT seeds germinated on MS medium or MS medium with mannitol (200 and 300 mM) or NaCl (150 mM) for 3 weeks.

Under normal conditions (Murashige and Skoog medium, MS medium), there were no obvious differences in seed germination and seedling growth between transgenic and wild type (WT) *Arabidopsis.* However, the *IpASR OX* plants grew better than the WT plants on MS medium supplemented with 200 mM mannitol or 100 mM NaCl (Figure 8A). They displayed larger cotyledons than the WT plants, and the seed germination rates of the transgenic lines were significantly greater than those of the WT plants, and corresponded with the increased mannitol (200, 300 and 400 mM) and salinity (150, 175 and 200 mM) concentrations (Figure 8B,C). When grown on MS medium containing different concentrations of mannitol (200 and 300 mM) or NaCl (150 mM) for three weeks, the WT and *IpASR OX* line seedlings exhibited an obvious difference (Figure 9). These results indicated that *IpASR* could enhance salt and osmotic stresses tolerance in transgenic *Arabidopsis* in both the seed germination and growth stages.

**Figure 10:**
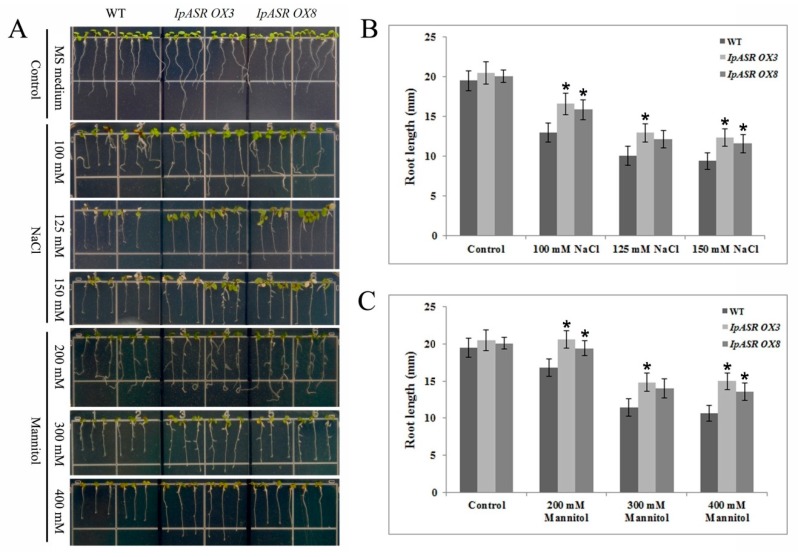
Osmotic and salt stress analyses of transgenic plants with *IpASR* based on seedling root length. Four-day-old seedlings were transplanted into MS medium containing NaCl or mannitol and then grown for 7 d before measuring the root length. (**A**) Photographs of transgenic lines (*IpASR OX3* and *IpASR OX8*) and WT seedlings on MS medium or MS medium with NaCl (100, 125 and 150 mM) or mannitol (200, 300 and 400 mM); (**B**,**C**) Seedling root length (mm) in WT and transgenic lines under NaCl (**B**) and mannitol (**C**) stress after 7 days. Error bars indicate the SD based on over three replicates (*n* ≥ 3). Asterisks indicate significant differences from the control (Student’s *t*-test *p* values, * *p* < 0.05.

To further test the salt and osmotic tolerance, 4-day-old seedlings were transferred to MS agar medium containing NaCl (100, 125 and 150 mM) or mannitol (200, 300 and 400 mM) and further cultivated for 7 days, following which the root lengths were measured. When grown on medium without NaCl or mannitol, no significant differences were observed between the WT and transgenic plants (*IpASR OX3* and *IpASR OX8*). In contrast, the root lengths of both sets of transgenic plants were significantly longer than the WT plants when grown on medium containing NaCl or mannitol, suggesting that the overexpression of *IpASR* enhances salt and osmotic tolerance at the seedling stage (Figure 10).

**Figure 11:**
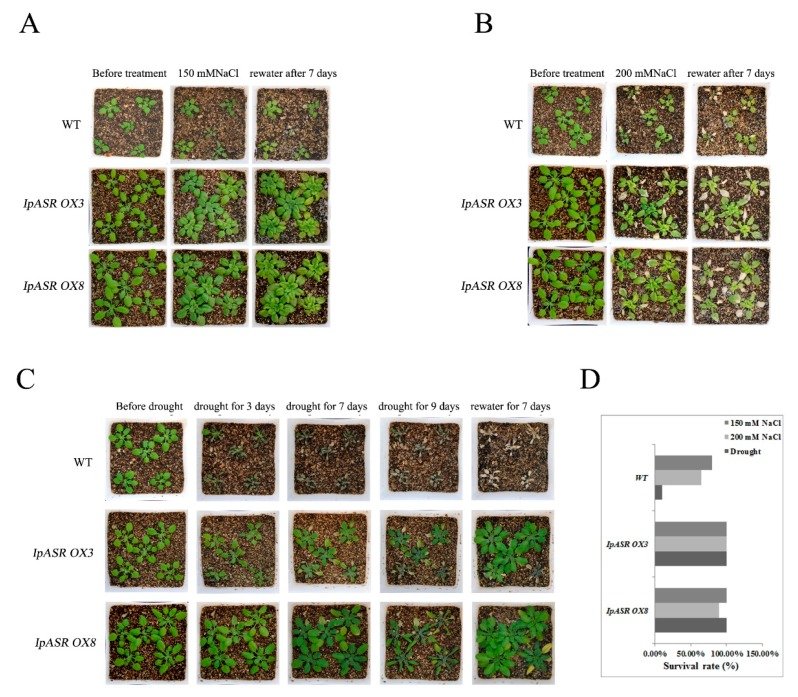
Photographs and survival rates of the transgenic overexpression lines and WT plants grown in pots under normal and salt/drought conditions. (**A**) The effects of 150 mM NaCl on transgenic lines and WT; (**B**) the effects of 200 mM NaCl on transgenic lines and WT; (**C**) the effects of withholding water on transgenic lines and WT. (**D**) The statistics for the survival rate of the transgenic lines and WT *Arabidopsis* after salt/drought stress. Thirty plants each of WT and two transgenic lines (*IpASR OX3* and *IpASR OX8*) were treated with various concentrations of NaCl or drought stress.

To further assess the function of *IpASR* during salt and drought tolerance, the corresponding treatments were applied to 3-week-old transgenic and WT plants grown under normal conditions with watering withheld for 10 days. The plants were then subjected to salt or drought treatment. At salinity stress levels of 150 and 200 mM NaCl, almost all the WT plants showed severely reduced growth, and even death, while the *IpASR OX* lines showed slight chlorosis and generally exhibited sustained growth and resistance to salinity stress (Figure 11A,B). Under normal conditions, there were no differences in the growth of the transgenic lines and the WT controls.

Additionally, the *IpASR* transgenic plants also showed characteristics of drought tolerance. The adult *Arabidopsis* plants (including *IpASR OX3*, *IpASR OX8*, and WT) for which watering was withheld for 10 days were primed for the drought-tolerance assay. After drought treatment, the majority of the WT plants exhibited obvious wilting due to severe dehydration, while the survival rates of the transgenic lines (*IpASR OX3* and *IpASR OX8*) were evidently higher than that of the WT controls (Figure 11C). After rewatering, the *IpASR OX* lines were largely restored, while the WT controls showed a lethal phenotype (Figure 11C).

The above results indicated that *IpASR* elevated the plant tolerance to salinity and drought stress and significantly improved the survival rates of transgenic *Arabidopsis* under osmotic or dehydration stress (Figure 11D). Furthermore, these findings indicate that the accumulation of IpASRs in transgenic *Arabidopsis* provides enhanced protection for basic cellular activities in vivo under osmotic stress.

### 2.8. Overexpression of IpASR Increases Relative Water Content (RWC) and Proline Content, and Decreases Ion Leakage (IL) and Malondialdehyde (MDA) Content under Osmotic Stress

**Figure 12:**
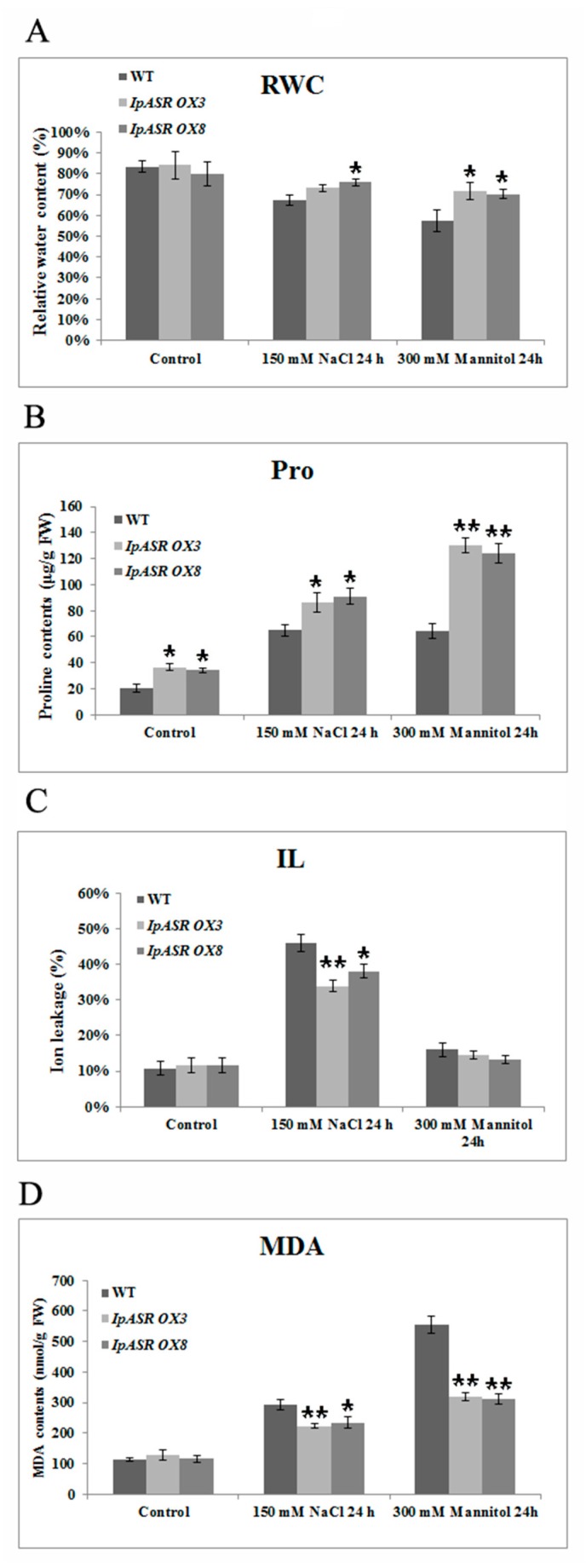
Changes in physiological parameters of *IpASR* overexpression *Arabidopsis* and WT seedlings (4 weeks) under 200 mM NaCl and 200 mM mannitol treatment for 24 h. (**A**) Relative water content (RWC); (**B**) Free proline content (Pro); (**C**) Ion leakage (IL); and (**D**) Malondialdehyde (MDA). Error bars indicate the SD based on over three replicates (*n* ≥ 3). Asterisks indicate significant differences from the control (Student’s *t*-test *p* values, * *p* < 0.05 and ** *p* < 0.01).

To further clarify the possible physiological mechanisms involved in cellular protection mediated by IpASR, several physiological indices, including RWC, proline content, ion leakage (IL), and MDA content, which are mainly related to cellular osmotic stress tolerance, were tested in the WT and *IpASR* transgenic *Arabidopsis* plants under salt and dehydration treatment. Compared to WT *Arabidopsis* plants, the RWC and proline content were higher in the transgenic lines when they were subjected to salt stress (150 mM NaCl, 1 day) or osmotic stress (300 mM mannitol, 1 day; Figure 12A,B), which indicated that the cells in the transgenic lines demonstrated better water status and stress resistance than the WT plants. IL, an important indicator of membrane injury, was higher in the WT plants than in the transgenic lines, suggesting that the transgenic plants suffered less membrane damage than the WT plants (Figure 12C). Accordingly, the MDA levels also displayed a pattern similar to IL, being lower in the transgenic lines relative to WT (Figure 12D). These physiological indices demonstrated that the transgenic lines were more resistant to salt and osmotic stress and indicated the protective roles of IpASR for the cellular membrane system, even for cell vitality under osmotic stress.

### 2.9. IpASR Overexpression Plants Resist Oxidative Damage by Increasing Reactive Oxygen Species ROS Scavenger Accumulation

**Figure 13:**
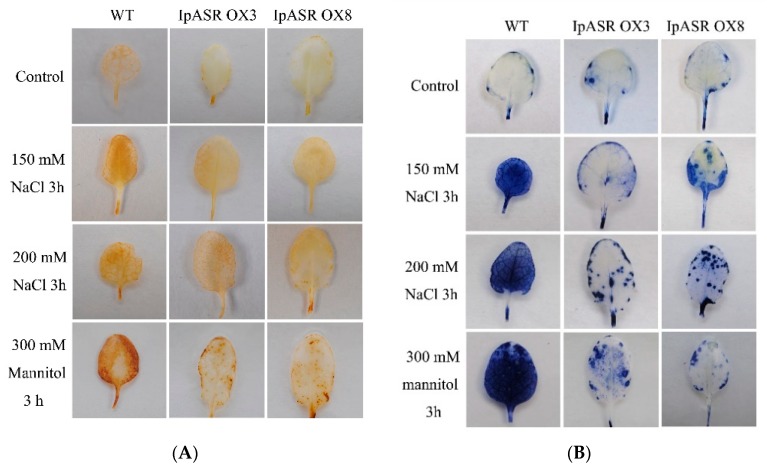

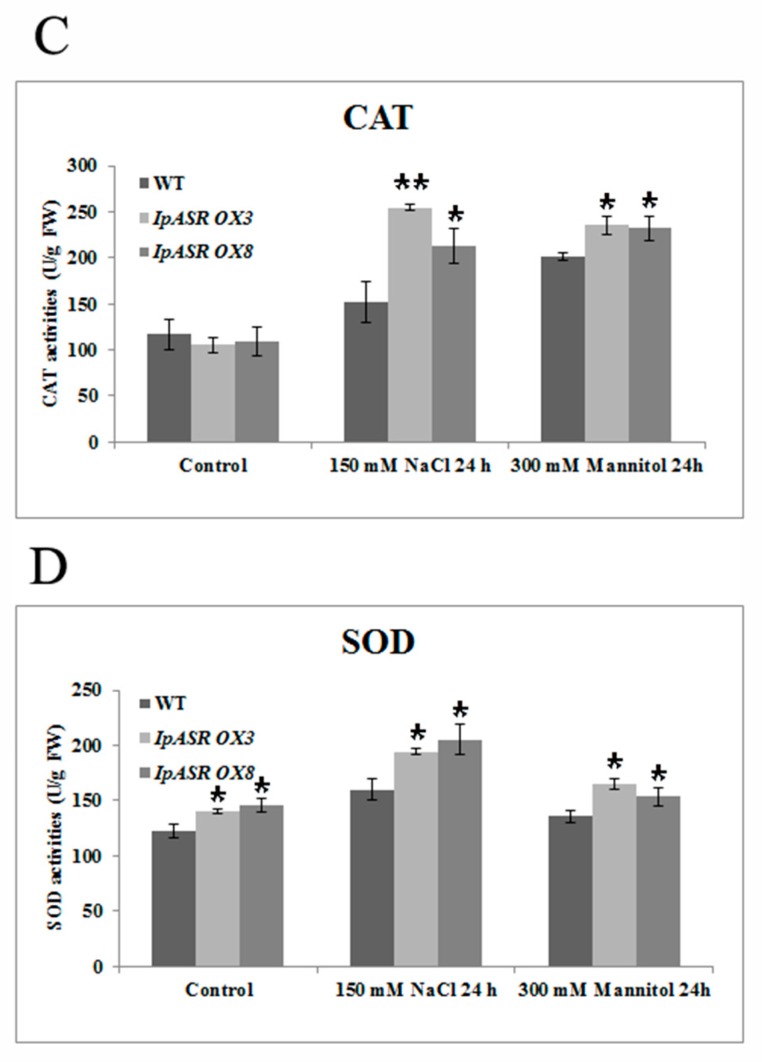
Oxidative stress analyses of transgenic overexpression lines and WT plants. Histochemical staining assays were used to detect H_2_O_2_ and O_2_^−^ by DAB (**A**) or nitro-blue tetrazolium (NBT) (**B**) staining, respectively. (**C**) Analysis of catalase (CAT) activity in the WT and transgenic lines under normal conditions and osmotic stress; (**D**) Analysis of superoxide dismutase (SOD) activity in the WT and transgenic lines under normal conditions and osmotic stress. Error bars indicate the SD based on over three replicates (*n* ≥ 3). Asterisks indicate significant differences from the control (Student’s *t*-test *p* values, * *p* < 0.05 and ** *p* < 0.01).

From Figure 12, it is evident that the MDA and proline contents differed between the WT and *IpASR* overexpression plants, which suggest that the cells in the *IpASR* transgenic plants showed stronger activity and better resistance to osmotic stress, including salt and drought. Proline and MDA are also important indices of plant oxidative stress and cell injury in response to stress conditions [20,21]. ROS are continuously generated as a consequence of abiotic stresses, such as drought, cold, salt and heat, causing a reduction in plant growth and crop yield losses worldwide [22]. When plant cells are subjected to stress, proline not only acts as an osmolyte, but also as a ROS scavenger [21], whereas the accumulation of MDA is often the final product of membrane lipid peroxidation caused by ROS accumulation. In other words, the differences in physiological indices between the WT and IpASR transgenic plants are eventually reflected in the cellular ROS accumulation and scavenging caused by osmotic stress. Therefore, it was essential that ROS accumulation be detected in the *IpASR* transgenic lines and WT. From Figure 13, the H_2_O_2_ and O_2_^−^ content increased both in the WT and *IpASR* transgenic lines after salt and mannitol (osmotic) stresses. The DAB (3,3′-diaminobenzidine) and NBT (nitro-blue tetrazolium) staining indicated that the WT plants accumulated more H_2_O_2_ and O_2_^−^ than the two *IpASR OX* lines (Figure 13A,B). Some enzymatic antioxidants, such as catalase (CAT) and superoxide dismutase (SOD), are believed to play significant roles in ROS scavenging and the cellular ROS balance. In this study, the activities of two significant antioxidant enzymes, CAT and SOD, were measured in the leaves from potted plants. Under normal growth conditions, the *IpASR* transgenic lines and WT plants showed some differences in CAT and SOD activities (Figure 13C,D). These results suggested that the overexpression of *IpASR* reduced ROS accumulation by enhancing SOD and CAT activities under osmotic stress.

**Figure 14:**
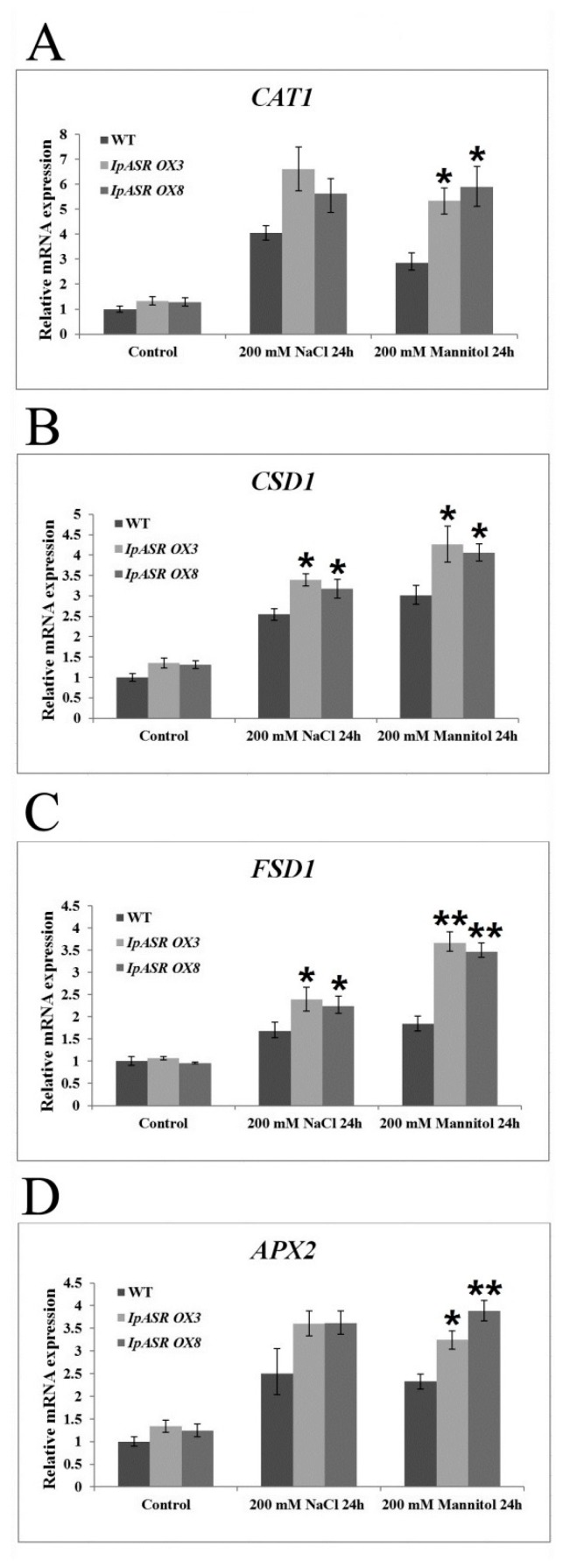
Analysis of the expression levels of reactive oxygen species- (ROS)-related genes in the WT and the transgenic overexpression lines by qRT-PCR under normal and osmotic stress conditions. (**A**) *CAT1*; (**B**) *CSD1*; (**C**) *FSD1*; and (**D**) *APX2*. Error bars indicate the SD based on three replicates. Asterisks indicate significant differences from the control (Student’s *t*-test *p* values, * *p* < 0.05 and ** *p* < 0.01).

To examine the possible molecular mechanisms underlying the function of *IpASR* in salt and dehydration resistance, the expression levels of several oxidation-related genes were analyzed by qRT-PCR in the *IpASR* transgenic lines and WT plants under 300 mM mannitol and 300 mM NaCl treatment (Figure 14). The four examined oxidative stress-responsive marker genes (*CAT1*, *CSD1*, *FSD1* and *APX2*) in *Arabidopsis* showed significantly upregulated transcription in the WT and *IpASR* transgenic plants under stress. This result suggested that IpASR accumulation in plants might upregulate the expression of these oxidative stress-responsive genes, and that these genes might improve plant resistance to salt and osmotic stress.

To further confirm the role of *IpASR* in regulating antioxidant mechanisms, the relationship between *IpASR* and photo-oxidative stress was also investigated by supplying MV to the *IpASR* transgenic and WT seedlings. MV is an herbicide that can generate highly reactive, oxygen-centered free radicals within chloroplasts when plants are exposed to sunlight [23]. As shown in Supplementary Materials Figure S1, when the plants were supplied with small amount of MV (20 and 50 μM, spray 3 times per pot ≈ 300 μL per pot), after 7 days, there is little differences between WT and *IpASR OXs*; while after increasing the amount of MV (both 20 and 50 μM) to 12-time spray (about 1.2 mL MV) per pot on the 7th day, after another 7 days, the WT leaves were bleached partly under 20 μM MV challenge, and showed almost withered under 50 μM MV challenge, while the color of the transgenic lines retained more green under the same concentration (Supplementary Materials Figure S1). Our results indicated that the *IpASR* transgenic lines demonstrated superior growth status under oxidative stress than the WT plant seedlings.

## 3. Discussion

Till now, only a few plant *ASRs* have nevertheless been characterized in terms of biological function, with most of them being involved in water deficit stress [4]. When plants suffer from water deficit (such as drought, freezing, high salinity, or ABA signaling caused by other stresses), *ASR* transcripts are induced, and a large amount of ASR proteins accumulate to reduce the cellular damage caused by the stresses, or to trigger the expression of some other stress response genes and the associated signaling pathways. The exact functions of ASR proteins remain unknown, but have been found to play an important role in improving the adaptability of plants to different abiotic stresses, including water deficit stress [3,24], as well as in regulating plant development and metabolism [4,25].

In the present work, a novel *ASR* gene, *IpASR*, was isolated and characterized from the extreme halophyte *I. pes-caprae* for the first time. Bioinformatics analyses indicated that IpASR is a highly hydrophilic, intrinsically disordered, and highly stable protein, possessing similar characteristics to LEA proteins. The ABA/WDS motif (PF02496) and other characteristics (Table 1) are regarded as key factors in reducing water loss in plants under stress conditions, which is equivalent to the role of hydration buffers in maintaining water balance [26,27,28]. Combining with the sequence alignment of different ASR members (Figure 1), we can infer that the ABA/WDS motif is highly conserved not only in sequence and secondary structure, but also in biochemical and biological functions, even till now there is no relevant reports about its specific functional mechanisms in plants.

Sequence phylogenetic analysis of IpASR and other plant proteins (Figure 2) showed that IpASR is highly homologous to InASR from morning glory (*Ipomoea nil*) [29], another Convolvulaceous plant. The expression of *InASR* in the corolla was upregulated along with flower senescence, while the transcription of *IpASR* also showed a high level of accumulation in the blooming flower of *I. pes-caprae*, indicating that it might be involved in the regulation of flower development and senescence as well as dehydration processes. Furthermore, IpASR also showed high homology with another two halophyte members, namely SbASR-1 (ACI15208.1) from *S. brachiate* [13,14] and SlASR (AGZ20206.1) from *S. liaotungensis* [15], both of which exhibited a strong positive correlation between transcript accumulation (overexpression assay in tobacco, groundnut, and *Arabidopsis*) and elevated resistance for salt and drought tolerance. The phylogenetic tree did reveal the close evolutionary relationship between three Convolvulaceous plant ASRs, including IpASR (*I. pes-caprae*), InASR (*I. nil*), and CsoASR (*C. soldanella*). However, due to the phylogenetic tree of ASR proteins was constructed based a few members, the evolutionary relationships of ASRs between glycophyte and halophyte, or monocot and dicot, could not be reflected in this tree. In addition, according to reports by Philippe et al. [30] and Cortés et al. [31], the diversity of the *ASR* genes was globally low in the context of drought tolerance, and different *ASR* members undertook different degrees of evolutionary and natural-selection pressure, subsequently presented different haplotype diversity or gene diversity. In summary, the above-mentioned plant ASRs are all involved in water deficit stress. Since the sequence homology usually reflects the functional similarities of different proteins, our bioinformatics analysis revealed that IpASR could be involved in the adaptation of *I. pes-caprae* to extreme saline and arid environments in tropical and subtropical coastal regions.

Salt, drought, and other abiotic stress factors ultimately result in the significant accumulation of ROS, thereby evoking the antioxidant system of plants [22]. Although there is a lot of evidence regarding the participation of plant ASRs in the response to abiotic stress, there is some direct evidence suggesting that plant ASRs have roles in the antioxidant activity of plants [4]. For instance, the overexpression of wheat *TaASR1* in tobacco enhances the expression of ROS-related and stress-responsive genes under osmotic stress, thereby improving the drought tolerance and MV resistance of tobacco [28]. The rice ASR1 can both scavenge ROS [32] and act as a transcription factor regulating aluminum responsive genes in rice [33]. A soybean ASR protein can bind metal ions and provide cellular antioxidant protection [34]. In our previous research, IpASR could elevate H_2_O_2_ tolerance when expressed in yeast (data not published), whereas in this report the induction of IpASR in *E. coli* resulted in the transgenic *E. coli* exhibiting higher tolerance than the control not only to NaCl and sorbitol, but also to H_2_O_2_. This further implies that the IpASR protein not only improves the resistance of *E. coli* to osmotic stresses via its highly hydrophilic ability, but at the same time also alleviates the toxicity of ROS to *E. coli* cells in an unknown manner, thus improving the antioxidant ability of *E. coli*.

Based on its physical and chemical properties, IpASR exhibits a heightened level of hydrophily, mainly mediated by high contents of hydrophilic amino acids, such as glutamic acid (17.67%), lysine (11.63%), and glycine (11.16%). Combined with the ABA/WDS domain, this feature ensures the functionality of IpASR in water deficit. Beside that there is still an important motif, designated as a histidine-rich motif (also named zinc-binding domain) at the N-terminal, which probably mediated the metal-binding (including Zn^2+^, Fe^3+^, Ni^2+^ and Cu^2+^) and in turn underwent conformational transition from unfolded to folded, then prevented the formation of ROS, or changed the protein’s solubility [7,34]. By analyzing the ASR sequences, we discovered that most ASRs at the N-terminal possess the His-rich motif, as does IpASR. Here we can speculate that this conserved His-rich motif might play some roles in cellular antioxidant responses when challenged by environmental stresses. However, there are still some other ASR members, such as two halophyte ASRs, namely SlASR from *S. liaotungensis* and *SbASR-1* from *S. brachiate* [13,14,15], which lack this typical motif. This further illustrates that there might be other mechanisms mediating the response of ASRs to ROS scavenging and abiotic stresses.

The transcription patterns of genes closely reflect the biological function of the genes. Our research indicated that the expression of *IpASR* was induced by high-salt (300 mM NaCl), osmotic (300 mM mannitol), oxidative stress (MV), low temperature (0 °C), and ABA treatment, which further indicated that *IpASR* is an important stress response gene in *I. pes-caprae*. Additionally, the IpASR protein is located in the nucleus, suggesting that this protein might be a chaperone or transcription factor in the cell nucleus that alleviates the harm of environmental stress to plant cells. We performed a transcriptional activity assay in yeast, which allowed us to speculate whether IpASR exhibits transcription factor activity and if the action domain is located at the N-terminal (Figure 6).

Over the past few decades, several model plants, such as tobacco and *Arabidopsis* as well as microorganisms, have been used to carry out the functional validation and characterization of genes from plants [13,15,16,35,36]. Here, we expressed IpASR in *E. coli* for the primary functional validation of IpASR in water deficit and anti-oxidation (Figure 4). To further clarify the function of *IpASR* under abiotic stress conditions, *IpASR* was overexpressed in *Arabidopsis* plants (Figure 7). Phenotypic analyses indicated that *IpASR* in *Arabidopsis* considerably enhances salt and osmotic resistance (Figure 8, Figure 9, Figure 10 and Figure 11) and also alleviates ROS toxicity (Figures 13 and Supplementary Materials Figure S1). ASR proteins play important roles in abiotic stress tolerance in most plants, as observed for SiASR4, TaASR1, OsASR1/5, and ZmASR1 [16,28,32,33,37]. Our research indicated that the overexpression of *IpASR* in *Arabidopsis* resulted in stronger tolerance to salt/drought in combination with reduced ROS accumulation and the elevation of the cellular antioxidative enzyme system. Here, we presumed that IpASR could act as a protective protein to maintain enzyme activities or to obstruct ROS production by binding to metals. Furthermore, IpASR exhibits transcription activity (Figure 6), which implies that the accumulation of IpASR affects the expression of some genes. We also assessed the transcripts of some antioxidative genes, and their expression patterns all changed both under control and stress treatments (Figure 12). This indicated that IpASR might act as a transcription factor and thus regulate gene expression, with surplus IpASR elevating plant abiotic stress tolerance.

In summary, a salt- and drought-related gene, *IpASR*, from *I. pes-caprae* was characterized in this research. Our results indicated that IpASR belongs to ABA/water-deficit stress-related category (PF02496, ABA/WDS motif). The expression of *IpASR* is induced by mannitol, high salinity, MV, cold, and ABA. The overexpression of *IpASR* in *E. coli* and *Arabidopsis* displayed complex expression patterns involved in responses to abiotic stress, primarily salt and drought tolerance. Nonetheless, *IpASR* acts as a downstream factor in the response of plants to salinity and drought stresses and may be an important candidate gene for the molecular breeding of salt-tolerant plants. Our findings indicated that IpASR acts as a transcription factor, hydrophilic protein, or an ROS scavenger; and has a pleiotropic effect on physiological processes, thereby improving plant tolerance to multiple abiotic stresses. This report should help elucidate the molecular mechanisms of IpASR from *I. pes-caprae* in salt and drought resistance, and provides valuable information for the development of crops with enhanced abiotic stress tolerance, ultimately aiding breeding programs aimed at improving salt/drought tolerance.

## 4. Materials and Methods

### 4.1. Plant Materials, Growth Conditions, and Stress Treatments

The *I. pes-caprae* plants were cultivated in the South China Botanical Garden (23°18′75.91″ N, 113°37′02.38″ E) in Guangzhou city. The *I. pes-caprae* seeds were collected from the seaside of Zhuhai city (22°16′25.37″ N, 113°34′18.00″ E), Guangdong province, China. Different parts of *I. pes-caprae* were frozen in liquid nitrogen and stored at −80 °C until RNA isolation.

For *I. pes-caprae* seedling culture, the seeds were sterilized with 70% ethanol and then the seed coats were broken with emery paper prior to being placed onto Murashige and Skoog (MS) basal salts distributed into plates with sand and soil, and seedlings grew outdoors from April to November (2016) in the South China Botanical Garden. The seedlings were used for stress treatment assays to assess the expression patterns of *IpASR*. Subsequently, salt (300 mM NaCl), simulated drought or osmotic (300 mM mannitol), oxidative (0.1 mM methyl viologen), and cold (0 °C) stresses and ABA treatment (0.1 mM) were applied to the *I. pes-caprae* seedlings to detect the expression patterns of *IpASR*.

*Arabidopsis thaliana* (ecotype: Col-0) plants used for the ectopic expression experiments were grown on solid MS medium for about 10 days before being transferred to soil. All plants were incubated in a growth chamber at 22 °C and a photoperiod of 16-h light/8-h darkness.

### 4.2. Isolation of the Full-Length IpASR cDNA

A full-length cDNA library from *I. pes-caprae* was constructed and screened with the Full-length cDNA Over-eXpressor (FOX) gene hunting system using a yeast salt-sensitive mutant (*AXT3*) complementary assay approach (data not published). Thereafter, a full-length cDNA encoding the ASR protein (IpASR) that rescued the phenotype of *AXT3* was selected and further investigated.

### 4.3. Sequence Analysis of the IpASR Gene

The full-length ASR cDNA sequence (GenBank accession no.: MF680587) was translated using the online ORFfinder translation tool (Available online: https://www.ncbi.nlm.nih.gov/orffinder/). The ABA/WDS domain (Pfam entry PF02496) of the ASR protein was identified using the Pfam 31.0 server (Available online: http://pfam.xfam.org/). The 3D prediction of IpASR was also conducted with the online program Protein Fold Recognition Server tool (PHYRE^2^) (Available online: http://www.sbg.bio.ic.ac.uk/phyre2/html/page.cgi?id=index). MEGA 6 was used for the protein homology comparisons and phylogenetic reconstruction using the neighbor-joining (NJ) method [38]. Bootstrap values were estimated (with 1000 replicates) to assess the relative support for each branch. The IpASR protein was aligned with known plant ASRs using ClustalW software (Available online: http://clustalw.ddbj.nig.ac.jp/).

The genomic sequence of *IpASR* was also amplified with primer pairs IpASRPEF and IpASRPER (Supplementary Materials Table S2). The genomic DNA of *I. pes-caprae* was isolated with HiPure SF Plant DNA Kits (Magen, Guangzhou, China). The PCR product was inserted in pGEM T vector (Promega, Shanghai, China) and sequenced.

The protein sequences used were as follows: *S. liaotungensis*: SlASR (AGZ20206.1); *S. brachiata*: SbASR-1 (ACI15208.1); *Camellia sinensis*: CsASR (AHJ09608.1); *Solanum chilense*: ScASR (CBY05857.1); *Mesembryanthemum crystallinum*: McASR (AAC14177.1); *Calystegia soldanella*: CsoASR (BAB19963.1); *Brachypodium distachyon*: BdASR1 (XP_003565133.2); BdASR2 (XP_003567508.1); BdASR3 (XP_003567509.1); BdASR4 (XP_003577811.1); BdASR5 (KQJ82606.1); soybean: GmASR1 (NP_001336496.1); GmASR2 (NP_001237487.1); GmASR3 (XP_003536424.1); *Ipomoea nil* (morning glory): InASR (BAF46301.1); grape: VvASR (AAK69513.1); rice: OsASR1 (BAG88534.1); OsASR2 (BAS76319.1); OsASR3 (BAG89007.1); OsASR4 (XP_015633592.1); OsASR5 (BAG99580.1); OsASR6 (BAG87564.1); Maize: ZmASR1 (NP_001105361.2); ZmASR2 (NP_001278619.1); ZmASR3 (NP_001278619.1); ZmASR4 (NP_001152067.2); ZmASR5 (NP_001106235.1); ZmASR6 (XP_008645776.1); ZmASR7-1 (ONM32036.1); ZmASR7-2 (ONM32035.1); ZmASR7-3 (ONM32037.1); tomato: SlASR1 (Q08655.1); SlASR2 (P37219.1); SlASR3 (P37220.2); SlASR4 (AAY98032.1); SlASR5 (XP_004237807.1); apple: MdASR1 (XP_008340103.1); MdASR2 (XP_008358291.1); MdASR3 (XP_008348767.1); MdASR4 (XP_008381833.1); MdASR5 (XP_008381469.1).

### 4.4. Bacterial Overexpression and Salt, Osmotic, Dehydration, and H_2_O_2_ Tolerance Assays in E. coli

To further confirm the biological function of IpASR, the GST-IpASR fusion protein was inductively expressed in *E. coli*. The coding sequence (CDS) of *IpASR* was PCR-amplified using the primer pair (IpASREPF and IpASREPR) listed in Supplementary Materials Table S2. The PCR fragments were subsequently inserted into the *Bam*HI site of pGEX 6p-1, followed by the GST-tag with the in-fusion technique (BD In-Fusion PCR cloning Kit, Takara Bio USA, Mountain View, CA, USA), thereby yielding the recombinant plasmid IpASR-pGEX 6p-1. The recombinant plasmid and empty vector pGEX 6p-1 (as a negative control) were then transformed into *E. coli* BL21 (DE3). A single colony was then inoculated in liquid Luria-Bertani (LB) medium and allowed to grow overnight at 37 °C with constant shaking at 200 rpm. Inoculum (1%) from the overnight-grown culture was added to fresh LB medium (100 mL) containing 100 μg/mL of ampicillin and allowed to grow at 37 °C and 200 rpm. Expression was induced at an OD_600_ of 0.5 by 0.2 mM isopropyl β-d-thiogalactopyranoside (IPTG), and the cells were allowed to grow at 30 °C for 4 h with constant shaking at 200 rpm. The induced bacterial cells were harvested by centrifugation at 6000 rpm for 10 min at 4 °C, and the protein profiles were examined by 12% SDS PAGE.

A spot assay of *E. coli* was performed to test the stress tolerance of the IpASR protein, with three replicates for each sample. To evaluate salt, H_2_O_2_, and osmotic stress tolerance, cell cultures of *E. coli* (IPTG induced) containing pGEX 6p-1/IpASR-pGEX 6p-1 were adjusted to OD_600_ 1.0 and then diluted serially (to 1:10, 1:100, and 1:1000). Two microliters of each sample was spotted onto the LB plates containing 0.2 mM IPTG and the stress component (5% or 6% NaCl, 2 M sorbitol, 5 or 8 mM H_2_O_2_). For the drought tolerance test, 1 mL OD-adjusted cell cultures in tubes were immediately placed in a 40 °C drying oven and maintained there for 4 h. Then, 100 μL liquid LB medium was added to the samples, which were then maintained at 37 °C for 1 h to recover. The samples were then diluted and spotted onto LB plates with 0.2 mM IPTG. The plates were incubated at 37 °C for 10 h. The bacterial colonies were counted (colony former unit, CFU) and the differences were analyzed.

We also performed a growth curve assay of recombinant *E. coli* in liquid LB culture to further confirm the functionality of IpASR. Briefly, 1 mL inoculum (OD_600_ value 1.0) was added to 10 mL LB medium (supplied with 0.2 mM IPTG) containing salt (3% or 4% NaCl), sorbitol (0.8 or 1 M), or H_2_O_2_ (0.7 or 0.9 mM) and incubated at 37 °C with shaking (180 rpm). The aliquots were removed from each treatment every 2 h from 2 to 12 h and absorbance (OD_600_) was measured. Abiotic stress (salt, osmotic and H_2_O_2_) tolerances were determined with respect to the control cultures (bacterial cells and empty vector pGEX 6p-1 controls).

### 4.5. Subcellular Localization Analysis

The CDS (without a termination codon) of the *IpASR* gene was amplified by PCR using the primer pair IpASRGF and IpASRGR (Supplementary Materials Table S2). The PCR product was inserted into the *Bam*HI site of the pUC/GFP vector to generate an IpASR-GFP in-frame fusion protein, following the in-fusion technique (Clontech, Mountain View, CA, USA), yielding the recombinant plasmid IpASR-pUC/GFP. After sequencing confirmation, the fusion construct and negative control (empty vector) were co-transfected into protoplasts (3 × 10^4^ protoplasts) using polyethylene glycol (PEG)-calcium solution (0.4 g·mL^−1^ PEG 4000, 0.2 M mannitol, 0.1 M CaCl_2_). After washing and resuspending in W5 solution (154 mM NaCl, 125 mM CaCl_2_, 5 mM KCl, 5 mM glucose, 2 mM MES, mesophyll protoplasts were incubated under white light for 12–18 h. Green fluorescent protein (GFP) fluorescence was visualized using a confocal laser scanning microscope (LSM, 510 META, Zeiss, Jena, Germany). The NLS-mCherry construction was co-transformed with the construction or empty vector as a nuclear localized marker.

### 4.6. Analysis of Transcriptional Activities in Yeast

The full-length CDS of IpASR and three truncated IpASRs were cloned into vector pGBKT7 (Clontech, Mountain View, CA, USA) for transcription activation analysis. These constructs along with the negative control pGBKT7 and pGBKT7-Lam plasmids, or positive control pGBKT7-53 plasmid, were transformed into *Saccharomyces cerevisiae* strain AH109 using the lithium acetate mediated method according to the Yeast Protocols Handbook (Clontech, Mountain View, CA, USA), and the vector pGADT7-T was co-transformed with all of the above constructs and positive/negative contorls (pGBKT7-53, pGBKT7, and pGBKT7-Lam). The yeast clones was cultured in liquid SD-2 medium to OD_600_ until 1.0, after which they were diluted using a gradient dilution (1:10, 1:100, and 1:1000). Two-microliter yeast cultures were spotted onto the corresponding synthetically defined (SD/-Trp/-Leu and SD/-Trp/-Leu/-His) medium plates for 2 days at 30 °C. Yeast transformation and determination of blue/white colonies were conducted according to the instructions of the manufacturer (Clontech), and X-α-Gal was used as a substrate for the reporter gene *MEL1*. Primers used for plasmid construction are shown in Supplementary Materials Table S2.

### 4.7. Expression Pattern Analysis

Total RNA was isolated from the *I. pes-caprae* tissues using HiPure Plant RNA Kits (Magen, Guangzhou, China), and the cDNA was synthesized by TransScript One-Step gDNA Removal and cDNA Synthesis SuperMix (TransGen Biotech, Beijing, China) according to the manufacturer’s instructions. Quantitative reverse transcript PCR (qRT-PCR) was performed to examine the expression levels of *IpASR* in the various tissues of the seedling and adult *I. pes-caprae* plants, including seedling roots, seedling leaves, buds, mature roots, vines, mature leaves, flower buds, petals, and young seeds. The *I. pes-caprae* seedling samples (roots, vines, and leaves), treated with salt, simulated drought or osmotic stress, oxidative stress, freezing stress, and ABA were also assessed to examine the expression changes of *IpASR*. All of the gene expression data obtained via qRT-PCR were normalized to the expression of *IpUBQ* (GenBank accession number: MF502417). The primers used for qRT-PCR are listed in Supplementary Materials Table S2.

For detection of the expression of antioxidative related genes (*CAT1*, *CAT2*, *CSD1*, and *FSD1*) in *Arabidopsis* (wild-type WT or transgenic plants), total RNA was isolated from the rosette leaves at different time points (with or without treatment), and cDNA synthesis was performed as per the above procedure. The reference gene for the RT-PCR was *ACT2* (At3g18780) in *Arabidopsis*. The primers used for qRT-PCR are listed in Supplementary Materials Table S2.

### 4.8. Generation of Transgenic Arabidopsis

To generate the recombinant vector for the overexpression assay in transgenic *Arabidopsis*, the full-length cDNA of *IpASR* was PCR-amplified using the primer pair IpASROXF and IpASROXR (Supplementary Materials Table S2). The PCR product was cloned into the *Bam*HI site of the pBIm plasmid [39] to generate IpASR-pBIm with an expression cassette under the control of the CaMV 35S promoter. After sequencing confirmation, the construct was transferred into *Agrobacterium tumefaciens* GV3101 and then transformed into *Arabidopsis* using the floral-dip method. Seeds of the T1 and T2 generations were screened on MS agar medium containing 50 mg/L kanamycin. Positive transgenic plants were selected according to the segregation ratio (resistant:sensitive = 3:1) and confirmed by genomic PCR with the primer pairs IpASROXF/IpASROXR. qRT-PCR and RT-PCR were also performed with primer pairs ASRF/ASRR and AtAct2F/AtAct2R (Supplementary Materials Table S2) in order to identify the expression level of *IpASR* in transgenic *Arabidopsis.*

### 4.9. Stress Tolerance of Transgenic Arabidopsis

For the salt and osmotic stress tolerance assay, transgenic *Arabidopsis* T3 (*IpASR OX3* and *IpASR OX8*) and WT were used. Sterilized seeds were germinated in MS medium under a 16-h photoperiod cycle at 25 °C, and 4-day-old seedlings were transplanted into medium supplemented with 100, 125, and 150 mM NaCl or 200, 300 and 400 mannitol. The germination rate and root length were measured. In brief, the seed germination rate of *IpASR* transgenic *Arabidopsis* was detected under NaCl (100, 125, 150, 175 and 200 mM) and mannitol (200, 300 and 400 mM) challenges on MS plates to assess whether the overexpression of *IpASR* could improve the salt and osmotic tolerance of the transgenic *Arabidopsis* seeds. The root length was also calculated to evaluate the influence of *IpASR* on the transgenic *Arabidopsis* seedlings under abiotic stress. The WT *Arabidopsis* was used as a control.

For salt and drought tolerance assays, 30 one-week-old seedlings of each genotype (*IpASR OX3*, *IpASR OX8*, and WT) were planted in sieve-like pots and watered well for two weeks. Prior to the salt/drought experiments, the plants of each genotype were cultured in a growth chamber as described above without watering for 10 days in order to ensure that the surrounding growing environment was devoid of excess water. The plants were then subjected to the following assays. For the drought tolerance assays, the WT and transgenic plants (*IpASR OX3* and *IpASR OX8*) were maintained under drought conditions for 9 days, and were then re-watered for 7 days. For the salt tolerance assays, the plants of each genotype were planted in sieve-like pots and watered well, as described for the drought tolerance treatment. Water was withheld for 10 d before irrigating with NaCl solution (150 and 200 mM) from the bottom of the plates. When the soil was completely saturated with salt water, the NaCl solution was removed and the plants were cultured normally. The plants were grown in the salt-saturated soil for 10 days and then re-watered for 7 days. The survival rate of the *Arabidopsis* plants was measured once the entire assay was complete.

For the oxidative stress analyses of the transgenic overexpression lines and WT plants, 3-week-old seedlings of *IpASR OXs* and WT *Arabidopsis* plants in the soil were sprayed evenly with 20 μM and 50 μM methyl viologen (MV) for two times (first plants were sprayed for 3 time at beginning each pot, then second session plants were sprayed for 12 time on the 7th day each pot, one spray ≈ 100 μL), after which the plants were cultured normally for 14 days. The phenotype was recorded.

### 4.10. Physiological and Histochemical Analysis

The relative water content (RWC) of adult *Arabidopsis* plants was determined as described according to Hu et al. [28]. Fresh weight (FW) of rosette leaves were recorded followed by soaking the leaves for 4 h in distilled water at room temperature with constant light. After this treatment the turgid weight (TW) was recorded. The leaves were then dried for 24 h at 80 °C to obtain the total dry weight (DW). RWC was calculated from the equation: RWC (%) = [(FW − DW)/(TW − DW)] × 100%.

Rosette leaves were collected after each treatment, and IL (ion leakage) was measured with a DDS-307A conductivity meter (Shanghai Jingke, Shanghai, China), according to Hu et al. [28] with slight modifications. *Arabidopsis* rosette leaves were incubated in 20 mL double-distilled water at room temperature with occasional shaking for 12 h. Initial conductivity (C1) was measured with a conductivity meter followed by boiling of the samples for 30 min to measure complete IL. The leaves were then cooled to room temperature to measure the electrolyte conductivity (C2). IL was calculated according to the equation: IL (%) = C1/C2 × 100%.

Free proline content, malondialdehyde (MDA) content, and superoxide dismutase (SOD) and catalase (CAT) activities were determined using proline, MDA, SOD, and Catalase Assay Kits, according to the manufacturer’s instructions, respectively (Nanjing Jiancheng, Nanjing, China). In situ detection of H_2_O_2_ and O_2_^−^ was determined with 1 mg/mL nitro-blue tetrazolium (NBT) or 1 mg/mL 3,3′-diaminobenzidine (DAB) solution for 12 h and clearing in 96% ethanol, respectively, as previously described [40].

### 4.11. Statistical Analysis

All the experiments in this study were repeated three times independently, and the data shown are the mean ± SD (*n* ≥ 3). Statistical analyses were performed using the statistical tools (Student’s *t*-test) of Microsoft Excel (Microsoft Corporation, Albuquerque, NM, USA).

## Figures and Tables

**Table 1:** The physical and chemical properties of the IpASR protein.

Physical and Chemical Properties	IpASR
No. of amino acids	215
Molecular weight (kDa)	24.57
Theoretical pI	5.42
Total no. of negatively charged residues (D + E)	49
Total no. of positively charged residues (R + K)	31
Grand average of hydropathicity (GRAVY)	−1.637
instability index (II)	36.82
Proportion of disordered amino acids (%)	77

## Supplementary Materials

The following are available online at http://www.mdpi.com/1422-0067/19/8/2252/s1.

## Abbreviations

ASR	Abscisic acid, Stress and Ripening
ABA/WDS	Abscisic acid/Water Deficit Stress
LEA	Late Embryogenesis Abundant
ORF	Open Reading Frame
GRAVY	Grand Average of Hydropathicity
IDP	Intrinsically Disordered Protein
NLS	Nuclear Localization Signal
MV	Methyl Viologen
IPTG	Isopropyl β-d-thiogalactopyranoside
LB	Luria-Bertani
GFP	Green Fluorescent Protei
MS	Murashige and Skoog
ROS	Reactive Oxygen Species
SDS PAGE	Sodium Dodecyl Sulfate Polyacrylamide Gel Electropheresis
qRT-PCR	Quantitative Reverse Transcript Polymerase Chain Reaction
OX	Over eXpression
WT	Wild Type
MDA	Malondialdehyde
IL	Ion Leakage
RWC	Relative Water Content
SOD	Superoxide Dismutas
CAT	Catalase
