# Supplementary material for: Ipomoea pes-caprae IpASR Improves Salinity and Drought Tolerance in Transgenic Escherichia coli and Arabidopsis
Source: Int J Mol Sci. 2018 Aug 1;19(8):2252. doi: 10.3390/ijms19082252 (PMC6121548; doi:10.3390/ijms19082252)

Supplementary Materials Information

Supplementary Materials Table S1. The sequences of *IpASR* cDNA, protein and genomic DNA (the red sequence indicated the CDS and the purple sequence indicated the intron).

cDNA sequence:

AACATATTTTAGCTTAGCATTCATATTTCGTCCACTACCAGAAACCATCCCAAAA
AACTAAAATAATCATGTCTGAGATGAAACACCACCACCACTTTGGTCACCACAA
AGACAATGAGGAAGAGAGGCAGTCTTCCTGTGGTGAAAACACTTATGGCACTGA
TGAGAAACCTTATGGCCAAACAGGCTATGGGGAAGAGTCTTATGAGAGGAAAA
ACACCTATGGTGATGACTCTTATGAGAGGAAAAACACCTATGGAGATGACTCTT
CTTGTGAGAGGAAAAACACCTATGGAGATGACTCTTCTTATGAGAGGAAAAACA
CTTATGGAGATGACTCTTATTATGAGAGGAAAAACACTTATGGAGATGACTCTTA
CGGCCAAATCGACAAGTATGGGAGTGAAGGCGTGACCGGCGGCATAGAACCGG
AAGGGAAAACTCATGAGGATTACGAAAAGGAGAAAAAACACCACAAGCATCTC
GAGCAGCTCGGCGGGCTTGGCACCGTCGCCGCCGGTGCCTATGCCTTGTACGAG
AAGCACGAGGCAAAGAAAGACCCAGAGAATGCGCACAAGCATAAGATAGCAG
AAGAGGTGGCGGCAGTGGCTGCCGTTGGATCAGGTGCATTTGCATTCCATGAGC
ATCATGAGAAGAAGGAAGCTAAGGAGGAAGAAGAAGAGGCTGAGGGAAAGAA
GAAGCATCACTTCTTTTAATTACATACTATATATGTGTTTTTATTATTAAGATCAA
ACTAAATAATCCGATCCTTAGCTTGTACGACGTGTGTGAATAATGGGTTTGGGCT
TATGCGTGTTGAGGTACGAGGAGGGTTTCTTTGTACCTAGCTAAGCTACCTATCT
ACTCTATTTGTGTCGACTGAGATGTTTTATTATTCTCTAGTGTATGTGGATTGTAAT
AATACTAATACTGATATAAAGTTTATTAAAAAAAAAAAAAAAA

Amino acid sequence:

MSEMKHHHHFGHHKDNEEERQSSCGENTYGTDEKPYGQTGYGEESYERKNTYGDDS
YERKNTYGDDSSCERKNTYGDDSSYERKNTYGDDSYYERKNTYGDDSYGQIDKYGSE
GVTGGIEPEGKTHEDYEKEKKHHKHLEQLGGLGTVAAGAYALYEKHEAKKDPENA
HKHKIAEEVAAVAAVGSGAFAFHEHHEKKEAKEEEEEAEGKKKHHFF

Genomic DNA sequence:

ATGTCTGAGATGAAACACCACCACCACTTTGGTCACCACAAAGACAATGAGGA
AGAGAGGCAGTCTTCCTGTGGTGAAAACACTTATGACACTGATGAGAAACCTTA
TGGCCAAACAGGCTATGGGGAAGAGTCTTATGAGAGGAAAAACACCTATGGTG
ATGACTCTTATGAGAGGAAAAACACCTATGGAGATGACTCTTCTTGTGAGAGGA
AAAACACCTATGGAGATGACTCTTCTTATGAGAGGAAAAACACTTATGGAGATG
ACTCTTATTATGAGAGGAAAAACACTTATGGAGATGACTCTTACGGCCAAATCG
ACAAGTATGGGAGTGAAGGCGTGACCGGCGGCATAGAACCGGAAGGGAAAACT
CATGAGGATTACGAAAAGGAGAAAAAACACCACAAGCATCTCGAGCAGCTCGG
CGGGCTTGGCACCGTCGCCGCCGGTGCCTATGCCTTGGTAATTTATTTTTATTTGA
CTTATGTAGTGATGAGTATGATAGCCTAATTGAAAAATCTTGGTGCAATATTAGA

ATTTAACATGGTATCATAACTTATTTAAATTCTAACATATCTGATACTACATCATA
TGCATGTATGTATGATTTTGTTAGTAATGGTTTTGGTTATTGGATGGGATGGATGG
ATGCAGTACGAGAAGCACGAGGCAAAGAAAGACCCAGAGAATGCGCACAAGC
ATAAGATAGCAGAAGAGGTGGCGGCAGTGGCTGCCGTTGGATCAGGTGCATTTG
CATTCCATGAGCATCATGAGAAGAAGGAAGCTAAGGAGGAAGAAGAAGAGGCT
GAGGGAAAGAAGAAGCATCACTTCTTTTAA

Supplementary Materials Table S2. Primer sequences used in this study.

Primer ID	Sequence (from 5′ to 3′)	Feature
IpASRPEF	GGGGCCCCTGGGATCCATGTCTGAGATGAAACACCA	gene primer pair for construction of IpASR-pGEX 6p-1, *Bam*HI site was underlined
IpASRPER	GGAATTCCGGGGATCCTTAAAAGAAGTGATGCTTCT	
IpASRGF	CTTGCTCCGTGGATCCATGTCTGAGATGAAACACCAC	gene primer pair for construction of IpASR-pUC/GFP, *Bam*HI site was underlined
IpASRGR	TGCTCACCATGGATCCAAAGAAGTGATGCTTCTTC	
IpASRRTF	AAGATAGCAGAAGAGGTGGCG	gene primer pair for qRT-PCR of *IpASR*
IpASRRTR	ACGCATAAGCCCAAACCCAT	
IpUBQRTF	TCGACAATGTGAAGGCAAAG	gene primer pair for qRT-PCR of reference gene *IpUBQ*
IpUBQRTR	CTTGATCTTCTTCGGCTTGG	
IpASRF1	CATGGAGGCCGAATTCATGTCTGAGATGAAACACCAC	primer pair for cloning the full-length CDS of *IpASR* and construction of pGBKT7-IpASR FL, *Eco*RI site was underlined
IpASRR1	GGATCCCCGGGAATTCAAAGAAGTGATGCTTCTTCTT	
IpASRR2	GGATCCCCGGGAATTCGGTGCCAAGCCCGCCGAGCTG	cloning the N-terminal of CDS for the construction of pGBKT7-IpASR N127 with IpASRF1, *Eco*RI site was underlined
IpASRR3	GGATCCCCGGGAATTCCAACGGCAGCCACTGCCGCCA	cloning the C-terminal of CDS for the construction of pGBKT7-IpASR N184 with IpASRF1, *Eco*RI site was underlined
IpASRF2	CATGGAGGCCGAATTCGATTACGAAAAGGAGAAAAAA	cloning the N-terminal of CDS for the construction of pGBKT7-IpASR C88 with IpASRR1, *Eco*RI site was underlined
IpASROXF	GGACTCTAGAGGATCCATGTCTGAGATGAAACACCA	for cloning the full-length CDS of *IpASR* and construction of IpASR-pBIm, *Bam*HI site was underlined
IpASROXR	GTCGACCCGGGGATCCTTAAAAGAAGTGATGCTTC	
ASRF	GCCAAACAGGCTATGGGGAA	gene primer pair for qRT-PCR of *IpASR* in transgenic *Arabidopsis*, or genomic DNA detection in *Arabidopsis*
ASRR	TTTGCCTCGTGCTTCTCGTA	
AtAct2F	GGTAACATTGTGCTCAGTGGTGG	gene primer pair for qRT-PCR of reference gene *AtActin2*
AtAct2R	AACGACCTTAATCTTCATGCTGC	

		(At3g18780) in *Arabidopsis*
CAT1RTF	CGCCATGCCGAAAAATACCC	gene primer pair for qRT-PCR of
CAT1RTR	CTTGCCTGTCTGAATCCCAGGAC	*CAT1* (At1g20630) in *Arabidopsis*
FSD1RTF	GCTCGGCTCTTTCCCATTGC	gene primer pair for qRT-PCR of
FSD2RTR	CAGCTTCCCAAGACACAAGATTG<br>G	*FSD2* (At4g25100) in *Arabidopsis*
CSD1RTF	TGATGGAACTGCCACCTTCACA	gene primer pair for qRT-PCR of
CSD1RTR	ATGGCCTCCCTTTCCGAGGT	*CSD1* (At1g08830) in *Arabidopsis*
APX2RTF	GGAAGCTCCGTGGTCTTATT	gene primer pair for qRT-PCR of
APX2RTR	CTCCTGTCTTCGTCTTCACATC	*APX2* (AT3G09640) in *Arabidopsis*

Supplementary Materials Figure S1. Oxidative stress analyses of transgenic overexpression lines and WT plants. Phenotypes of 3-week-old *IpASR OXs* and WT plants were treated with 20 and 50 μM MV for 14 d.

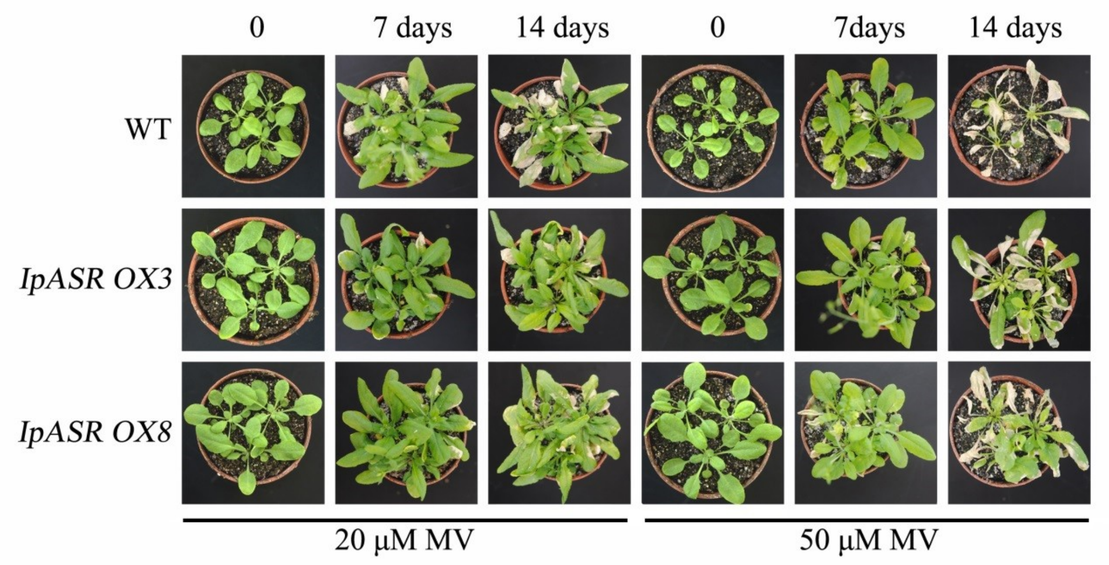

Supplement: Supplementary file 1 [file ijms-19-02252-s001.pdf]
